# Supplementary material for: Lexical Landscapes as large in silico data for examining advanced properties of fitness landscapes
Source: PLoS One. 2019 Aug 12;14(8):e0220891. doi: 10.1371/journal.pone.0220891 (PMC6690511; doi:10.1371/journal.pone.0220891)
Supplement: S1 Table — (a) and (b) show the number of 1-grams of each type gathered from the All English and British-English Googled Ngram Databases for this study, as well as the possible associated Lexical Landscapes that could be constructed from each set. S1 Table (c) and (d) likewise show the number of available words and the total possible landscapes that could be constructed from the reduced data sets used within this investigation. The reduced data sets were constructed using cutoffs in order to eliminate words with excessively low usage counts and avoid the presence of acronyms within Lexical Landscapes. (PDF) [file pone.0220891.s002.pdf]

	Available Words	Possible Landscapes
**3-letter**	17,338	7,665,017,103
**4-letter**	163,809	684,247,229,136
**5-letter**	419,185	4,480,748,948,520
**Total**	600,332	5,172,661,194,759

(a) All English

	Available Words	Possible Landscapes
**3-letter**	16,375	6,837,168,375
**4-letter**	111,675	318,015,445,725
**5-letter**	267,943	1,830,726,174,303
**Total**	395,993	2,155,578,788,403

(b) All British-English

	Available Words	Possible Landscapes
**3-letter**	200	1,014,900
**4-letter**	1,500	57,336,750
**5-letter**	5,000	637,372,500
**Total**	6,700	695,724,150

(b) Reduced English

	Available Words	Possible Landscapes
**3-letter**	250	1,587,375
**4-letter**	500	6,362,250
**5-letter**	5,000	637,372,500
**Total**	5,750	645,322,125

(d) Reduced British-English
