# Supplementary figures and images for: Lexical Landscapes as large in silico data for examining advanced properties of fitness landscapes
Source: PLoS One. 2019 Aug 12;14(8):e0220891. doi: 10.1371/journal.pone.0220891 (PMC6690511; doi:10.1371/journal.pone.0220891)

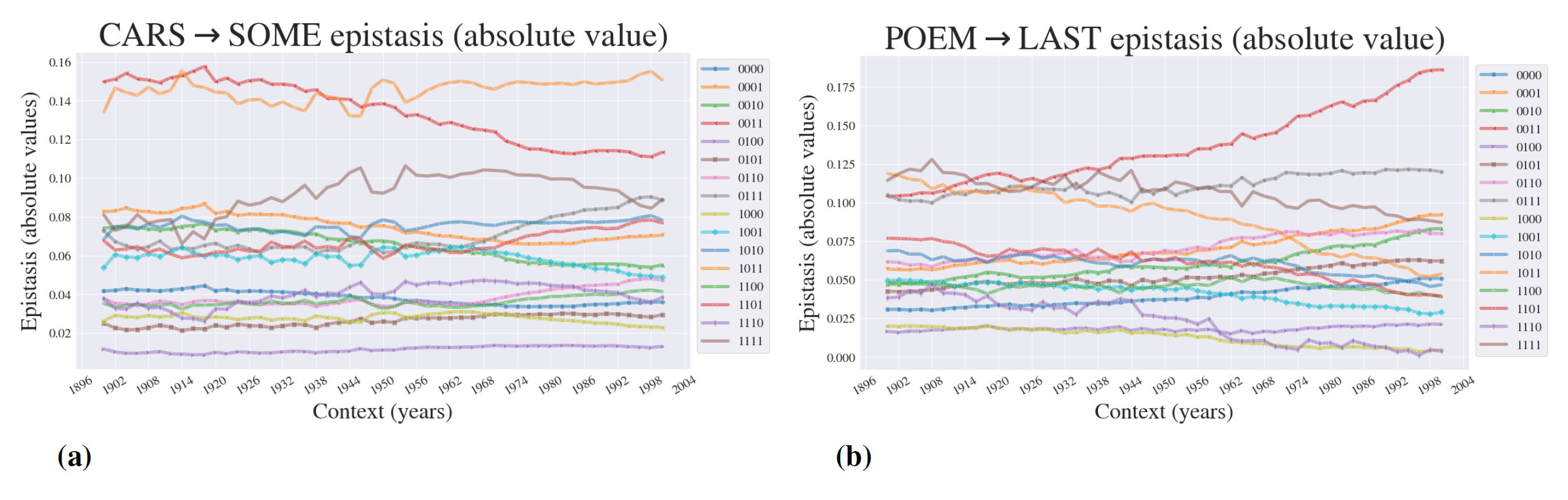

Supplement: S1 Fig — In the main text Fig 6 demonstrated the aggregated effects, the coefficients corresponding to 0th—4th order effects. S2(a) and S2(b) Fig show how individual epistatic effects can vary over time, or more generally, across context. Each line represents a particular epistatic effect, and the lines are grouped by order and demarcated by color theme and marker. For instance, the collection of light red to dark red lines shows the first order epistatic effects: 0001, 0010, 0100, 1000. (TIF) [file pone.0220891.s003.tif]

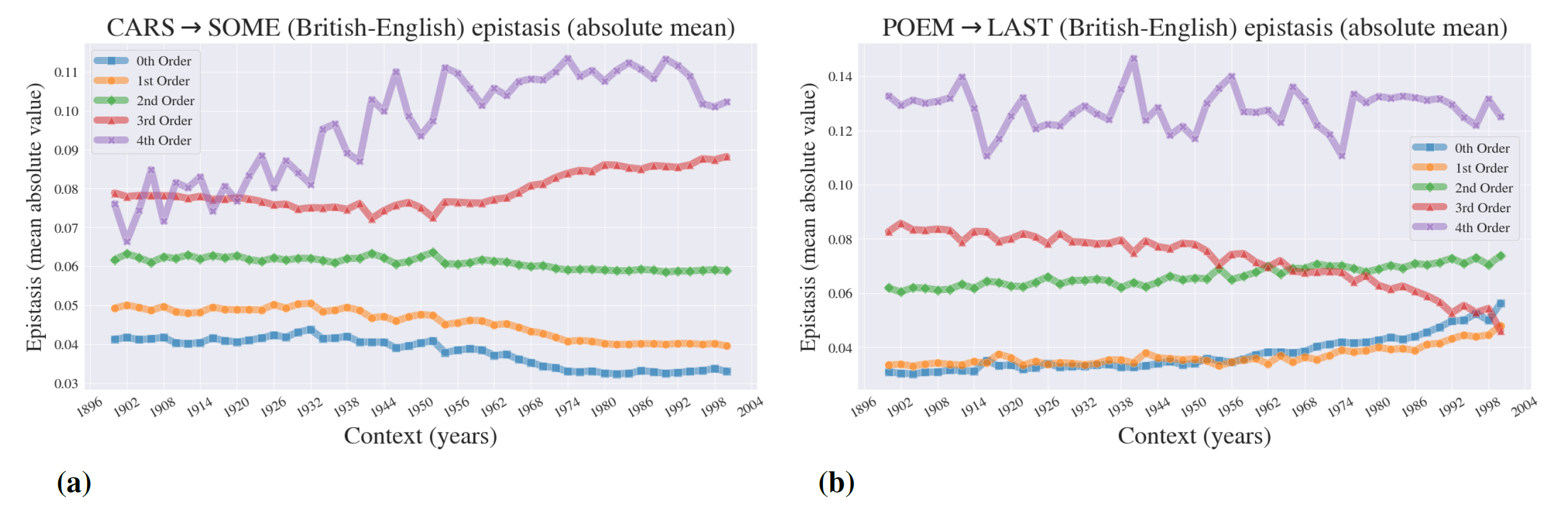

Supplement: S2 Fig — Fitness graphs for the POEM → LAST landscape. As described in this manuscript, the aggregated epistatic effects combine the averages of individual interactions and organize them by their order. S3 Fig shows these effects for the British-English CARS → SOME and POEM → LAST landscapes. (TIF) [file pone.0220891.s004.tif]

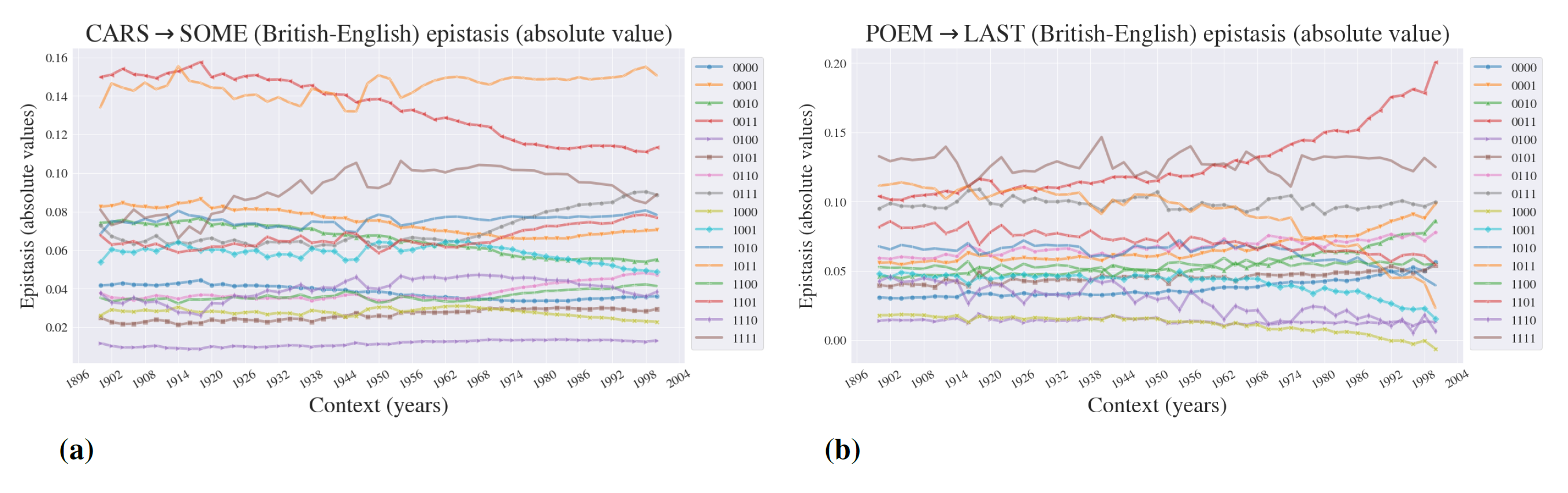

Supplement: S3 Fig — As discussed in several places throughout this manuscript, dis-aggregated graphs represent how individual epistatic terms interact across contexts. These graphs represent those effects for the CARS → SOME and POEM → LAST landscapes in British-English. (TIF) [file pone.0220891.s005.tif]

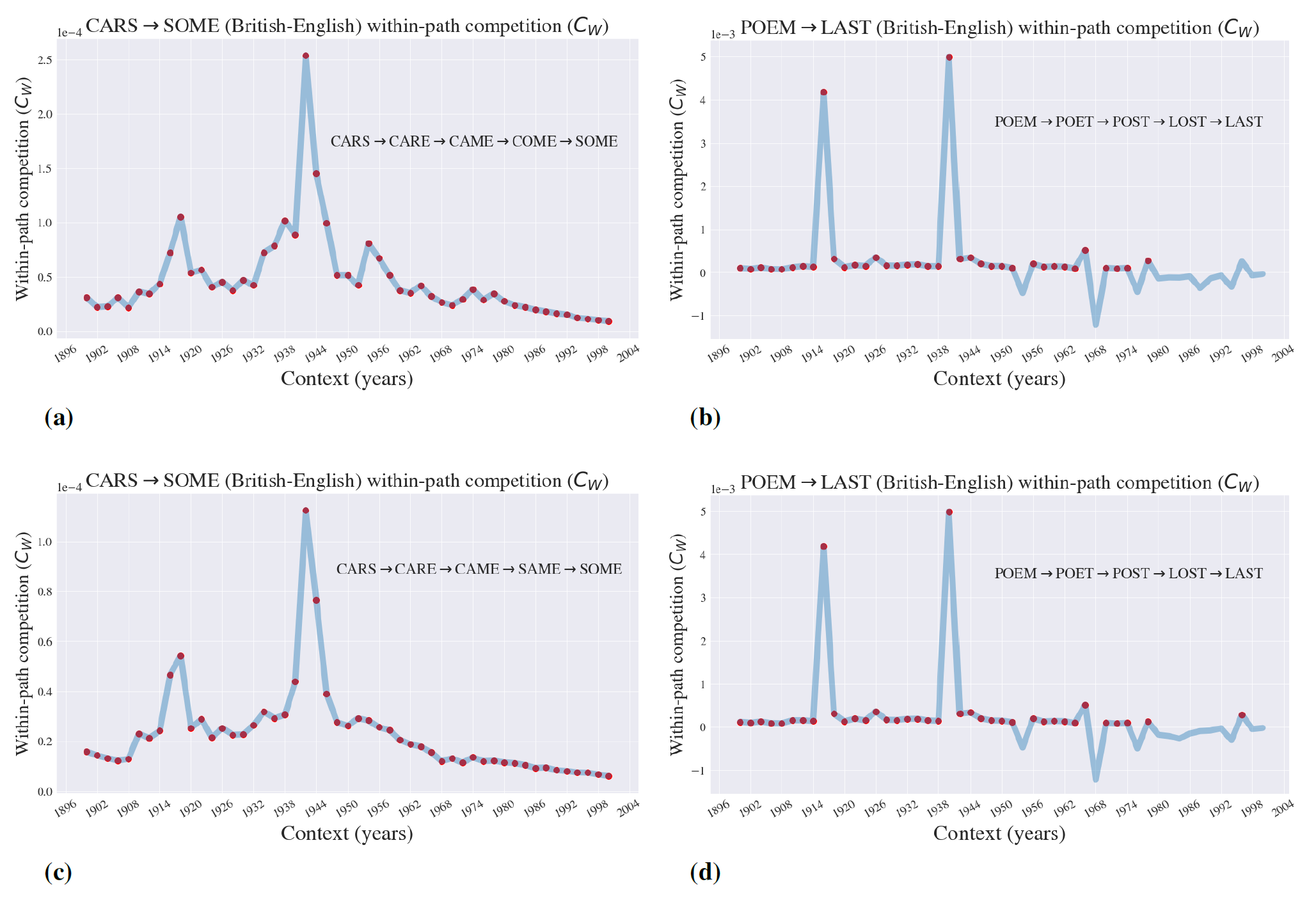

Supplement: S4 Fig — Here we present within-path competition in the British English Lexical Landscapes subset. (TIF) [file pone.0220891.s006.tif]

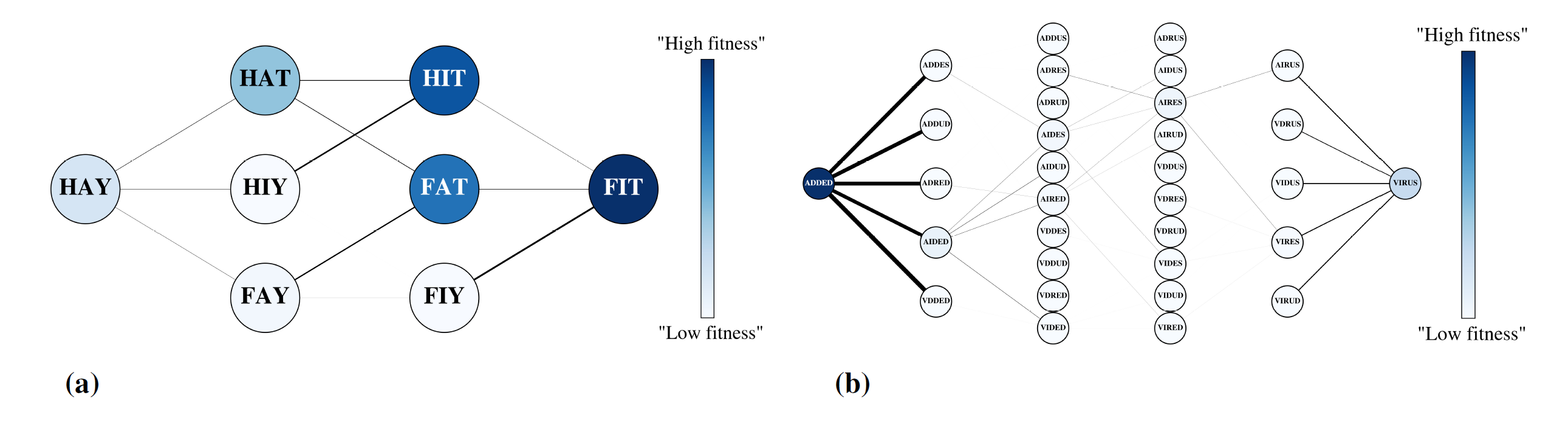

Supplement: S5 Fig — Fitness graphs for 3 letter (HAY → FIT) and 5 letter (ADDED → VIRUS) Lexical Landscapes. S6(a) and S6(b) Fig are visualizations of the fitness landscapes for three and five letters. The color indicates the fitness: darker the blue, higher the fitness. Edges in the graph are weighted by the difference (in absolute value) between the fitness values of the two adjacent nodes and are emboldened in a proportional way to show the weight. (TIF) [file pone.0220891.s007.tif]

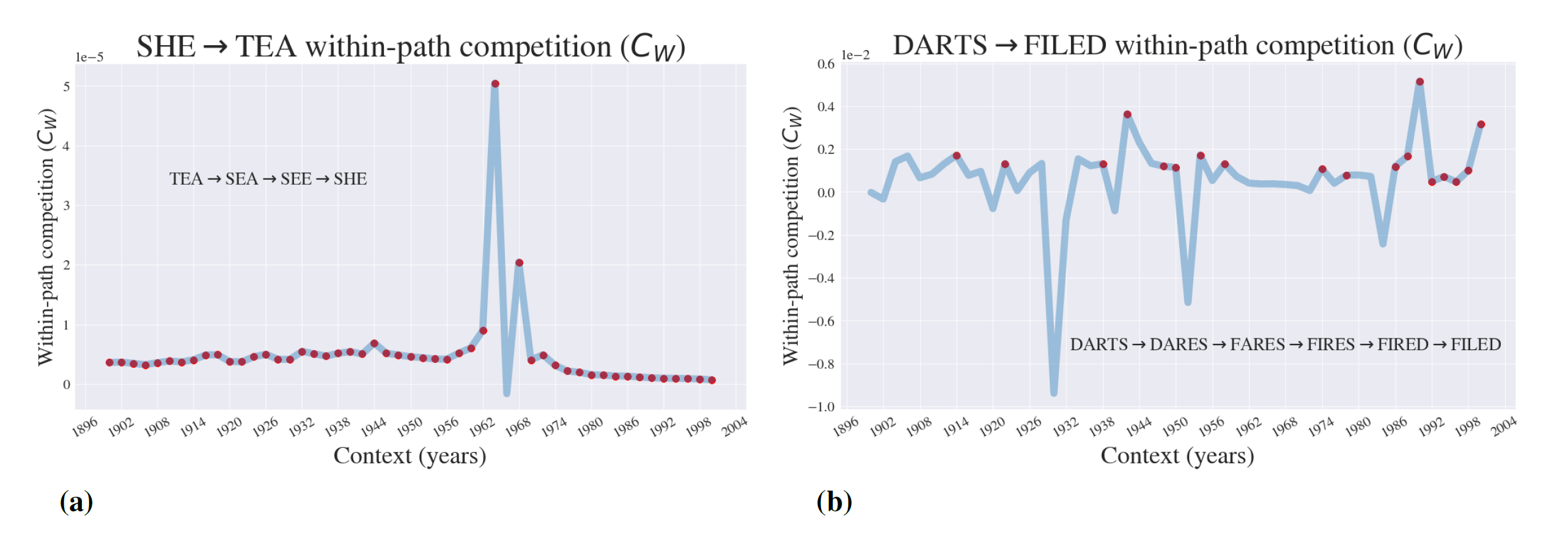

Supplement: S6 Fig — S7(a) and S7(b) Fig demonstrate that the (Cw) can vary across environment (as it did for the 4 letter 1-grams discussed in the main texts). Red dots indicate the years where the specified path—shown to the right of each figure—represented an uphill path in the fitness landscape, that is, a path where each successive word in the path has a higher fitness than the previous one. (TIF) [file pone.0220891.s008.tif]

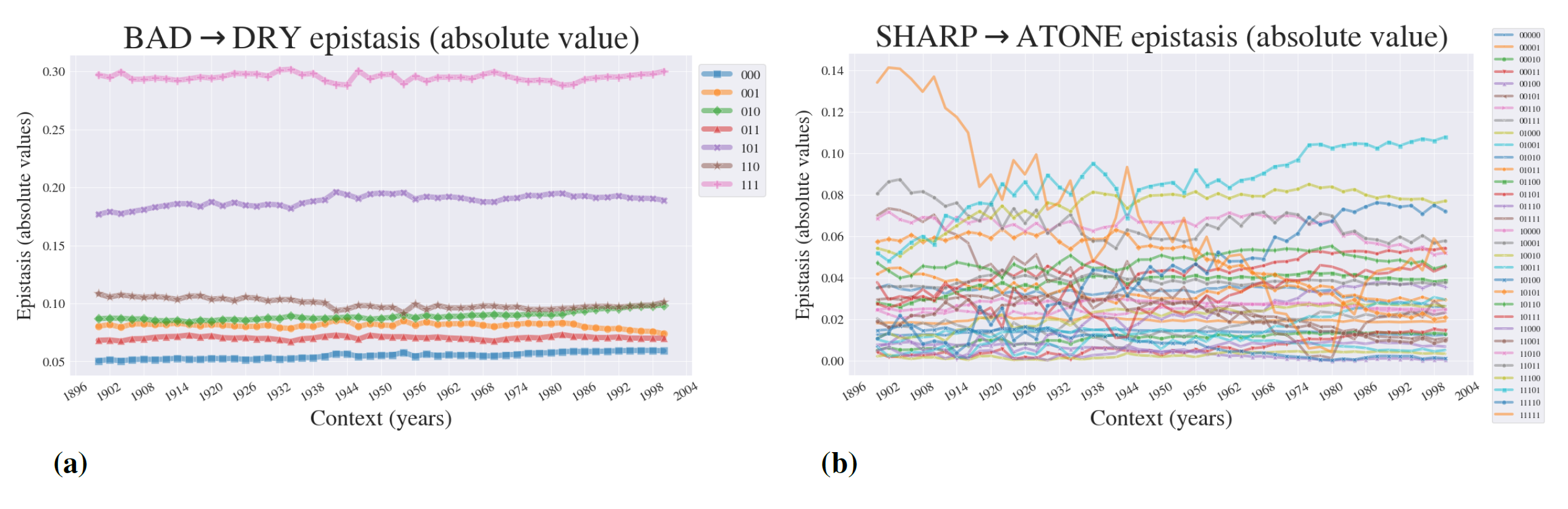

Supplement: S7 Fig — (a) and (b) show how epistatic effects for example three and five letter word landscapes can vary across environment, or in this case across time. We have chosen a three letter landscape (BAD to DRY) with mild fluctuations over time to contrast it with the relatively large fluctuations in the five-letter landscape (SHARP to ATONE). (TIF) [file pone.0220891.s009.tif]
